# Validation of a new hand-held electronic data capture method for continuous monitoring of subjective appetite sensations

**DOI:** 10.1186/1479-5868-8-57

**Published:** 2011-06-08

**Authors:** Catherine Gibbons, Phillipa Caudwell, Graham Finlayson, Neil King, John Blundell

**Affiliations:** 1 Biopsychology Group, Institute of Psychological Sciences, University of Leeds, Leeds, UK; 2 Institute of Health and Biomedical Innovation, Queensland University of Technology, Brisbane, Australia

## Abstract

**Background:**

When large scale trials are investigating the effects of interventions on appetite, it is paramount to efficiently monitor large amounts of human data. The original hand-held Electronic Appetite Ratings System (EARS) was designed to facilitate the administering and data management of visual analogue scales (VAS) of subjective appetite sensations. The purpose of this study was to validate a novel hand-held method (EARS II (HP^® ^iPAQ)) against the standard Pen and Paper (P&P) method and the previously validated EARS.

**Methods:**

Twelve participants (5 male, 7 female, aged 18-40) were involved in a fully repeated measures design. Participants were randomly assigned in a crossover design, to either high fat (>48% fat) or low fat (<28% fat) meal days, one week apart and completed ratings using the three data capture methods ordered according to Latin Square. The first set of appetite sensations was completed in a fasted state, immediately before a fixed breakfast. Thereafter, appetite sensations were completed every thirty minutes for 4h. An *ad libitum *lunch was provided immediately before completing a final set of appetite sensations.

**Results:**

Repeated measures ANOVAs were conducted for ratings of hunger, fullness and desire to eat. There were no significant differences between P&P compared with either EARS or EARS II (*p *> 0.05). Correlation coefficients between P&P and EARS II, controlling for age and gender, were performed on Area Under the Curve ratings. R^2 ^for Hunger (0.89), Fullness (0.96) and Desire to Eat (0.95) were statistically significant (p < 0.05).

**Conclusions:**

EARS II was sensitive to the impact of a meal and recovery of appetite during the postprandial period and is therefore an effective device for monitoring appetite sensations. This study provides evidence and support for further validation of the novel EARS II method for monitoring appetite sensations during large scale studies. The added versatility means that future uses of the system provides the potential to monitor a range of other behavioural and physiological measures often important in clinical and free living trials.

This study was registered as a clinical trial by Current Controlled Trials (Registration Number - ISRCTN47291569).

## Background

Visual Analogue Scales (VAS) have been used in clinical and research settings to continuously monitor a range of subjective sensations for example, pain, depression and appetite [1]. These measures provide valuable information on sensations that are difficult to monitor using alternative methods [2]. VAS typically take the form of 100 mm horizontal lines anchored at both ends by extreme subjective feelings [3]. This horizontal line represents a continuum and allows the participant to place a mark on the scale reflecting the intensity of a subjective sensation at a particular time (i.e. state). This allows the sensation to be measurable and quantifiable. The interpretation of VAS is simple since the descriptive terms are already present [1]. Traditionally, VAS were administered using pen and paper (P&P), which were quick and relatively easy to use. Data collection from P&P method is often time-consuming since each line needs to be measured and manually inputted into a spreadsheet individually introducing the possibility of human error. Whilst the use of P&P method has limited flaws when the participants are closely monitored, in free-living situations P&P method has considerable limitations for example when unsupervised, compliance is low [4] and questions may be omitted, wrongly marked or not filled in at the correct time resulting in invalid data [5].

To diminish the problems of using P&P, a step was taken to develop the same system of VAS on portable handheld computers, which became the appropriately named Electronic Appetite Ratings System (EARS). The transition to the use of handheld computers was driven by their relatively inexpensive cost and their associated practical benefits [6]. Additional benefits of electronic versions include the use of an audio alarm as a reminder of when the VAS needed to be completed, leading to compliance rates of 90% or better [7]. All entries are date- and time-stamped. The first EARS to be developed used a VAS software program which was designed to administer VAS using a Psion hand-held personal digital assistant (PDA) (^©^University of Leeds, UK). The software employed a 100 mm horizontal line with a vertical marker present at the mid-point. The arrow keys are used to move the cursor left or right to a particular position. A pilot study was conducted to compare the EARS with the standard P&P method [8]. Two different energy pre-loads were used to manipulate subjective appetite sensations. The results demonstrated that the mean area under the curve (AUC) ratings were similar between the two techniques, however there were significant differences between the techniques when some of the ratings at individual time points were compared (i.e., immediately before and after meals). Both techniques detected a significant difference between the high and low energy lunches [8]. The use of EARS was later compared to P&P in a clinical population of haemodialysis patients. For all questions, there was a bias towards lower scores on the EARS than P&P. Due to the unsystematic pattern of variation in the data and the high standard deviations giving wide limits of agreement, these factors suggest that there is limited amount of agreement between the methods and they therefore should not be used interchangeably [9].

Using a similar methodological approach, an alternative EARS was developed using the Apple Newton Message Pad, (^©^RJ Stubbs and M Elia) to administer the VAS. The main development was that participants used a 'stylus' to mark their responses on the screen of the Apple Newton - which is more akin to placing a mark on a paper VAS using a pencil. Two validation studies were conducted using EARS Newton to compare with the P&P [5,10]. The first compared the response of subjects (10 men, 10 women) after consuming meals at fixed time points. The meals were of fixed energy density, energy and nutrient content. The participants completed hourly VAS using both methods on day 1, which was then repeated after two intervening days (i.e., on day 4). The second study examined validity and reliability in a free-living environment rather than the laboratory. Both studies demonstrated that the temporal pattern of oscillations in subjective appetite sensations and the sensitivity remained similar. However, there were significant differences in hunger and fullness ratings at the extremes of the VAS, with the EARS Newton resulting in more constrained values than P&P. Bland and Altman analysis revealed that the mean difference between the two methods was significantly different to zero due to higher variance seen in the P&P method than EARS Newton. In the test-retest studies there were no significant differences in individual hourly ratings or AUC between the two techniques. Since both studies show the same differences between P&P and EARS Newton, and the same reliability, the authors concluded that EARS Newton is a valid method for administering VAS, both in controlled and free-living settings but it is not interchangeable with P&P.

Whybrow et al, (2006) have recently developed the EARS Newton system to make use of a Palm-handheld computer. The VAS software was identical to the previous studies of Stubbs except the screen size and length of VAS displayed was different. This study included (10 men, 10 women) using a similar test day design to Stratton et al, (1998) as previously described. There was an interaction between gender and method. Women tended to rate their appetite sensations higher when using EARS Palm than P&P for 3 of the VAS questions (i.e., hunger, desire to eat and prospective consumption). There was a statistical bias between the methods for 5 VAS questions leading to the conclusion that EARS Palm showed sensitivity and similarities to P&P, but cannot be used interchangeably.

Due to a series of technical issues and a halt in their manufacture, the Psion PDA has become obsolete. Using a similar approach, new software was developed for a different EARS (HP iPAQ 214) (^©^Queensland University of Technology (QUT)). The aim of the present study was to validate the new EARS II against the gold standard P&P and the previously validated EARS in standardised conditions.

## Methods

### Participants

Twelve participants were recruited and gave their informed consent to this study (5 male, 7 female). One participant failed to complete the evaluation questionnaire. Subjects characteristics (±s.d) were mean age 25.6yr (±6.3yr), mean weight 68.65 kg (±13.48 kg), mean height 1.71 m (±0.09 m) and mean BMI 23.62 kg/m^2 ^(±2.71 kg/m^2^). Ethical permission to carry out this study was granted by the Institute of Psychological Sciences Ethics Committee, University of Leeds. Volunteers were recruited from the university and consisted of both students and members of staff. Volunteers were recruited via posters and word-of-mouth. All participants gave their written, informed consent. This study was also registered as a clinical trial ISRCTN47291569.

### Design

The study employed a within-subject crossover design - with method (3 - P&P, EARS and EARS II), meal (2 - HF and LF) and time (11 - time points) as the independent factors. All participants took part in two identical study days using both high- and low-fat meal manipulations.

### Measurement Day

Participants were assigned to high fat (>48% energy from fat) or low fat (<28% energy from fat) meal days in a counterbalanced order with 7-10 days between. Appetite ratings consisted of the questions regarding 'hunger', 'fullness' and 'desire to eat'. The first set of ratings was completed in a fasted state, after which a fixed breakfast was provided. VASs were completed every thirty minutes for 4h. An *ad libitum *high- or low-fat lunch was provided 4h after the fixed breakfast. Immediately after consuming the lunch participants completed a final set of ratings. Participants were reminded and instructed by the researcher when to complete the P&P method, the EARS and the EARS II. Each method was completed immediately after one another, in alternating sequence determined by Latin Square. The participants remained in a cubicle in the laboratory for the morning so that their VAS ratings and food consumption were unaltered by extraneous factors. During their free time, they were permitted to use computers, read etc. within the laboratory.

### Appetite Ratings

Instructions regarding the use of all three methods were given verbally during an allotted practice period. Participants were told to consider the extremes of each rating as the most intense sensation they can imagine. Participants were presented with a series of questions accompanied by horizontal lines anchored at each end by the words "Not at all" and "Extremely". The wording and order of questions was identical for each of the three methods and included "How hungry do you feel now?"; "How full do you feel now?"; "How strong is your desire to eat now?". The P&P method involved marking a single vertical line using a pencil. Once completed the P&P VAS were removed from the sight of the participant to ensure they could not refer back to previous answers.

The EARS method involved a small hand-held computer with questions presented on screen one at a time. Participants were asked to read the question and then use the arrow keys to move a centred vertical cursor along the horizontal line. Once the cursor is in place, the participant was instructed to press 'enter', and then confirm the response. If the participant had made a mistake, then it could be corrected at this point.

Finally the EARS II method was similar to EARS in that one question was viewed at a time. The horizontal dimension of the VAS scales were 84 mm, with the horizontal line being 100 pixels in length. Therefore, the VAS scale is 0-100 units. Participants read the question then used a stylus to mark their response along a horizontal line. The participants were informed that the first touch of the stylus would make a vertical marker appear and that the stylus could then be used to move this marker along the horizontal line. Once the marker was in place, the participant pressed a 'continue' box at the bottom of the screen and then confirmed the response to proceed. If the participant made a mistake, they were instructed to inform the researcher who would ensure the question was repeated.

### Fixed Breakfast

Participants arrived at the human appetite research unit at ~0800 after an overnight fast. On both measurement days, participants were instructed to eat everything provided at the fixed breakfast meal consisting of standard breakfast products which were all commercially available. The fixed breakfast meal consisted of cereal with milk, toast with scrambled egg, margarine, sugar and tea/coffee. The mean energy content provided was 538kcal (2252kJ) on the HF day and 551kcal (2307kJ) on the LF day. These meals consisted of the same weight of food (318 g) and the relative contribution of energy from fat, protein and carbohydrates were 42.7%, 14.6% and 42.7% respectively on the HF day and 27.3%, 16.3% and 56.4% on the LF day.

### Food Intake

After the fixed breakfast, food intake was directly measured using an *ad libitum *lunch at 4h post-breakfast. The lunch meals (average of whole meal) were manipulated to be HF with 54% energy from fat, 12% from protein and 34% from carbohydrate and LF with 27% energy from fat, 14% from protein and 59% from carbohydrate. The meal provided at the lunch consisted of three rounds of cheese sandwiches, 100 g crisps and 100 g chocolate snack, each of a high or low fat variety. The participant was instructed to eat until comfortable fullness.

### Evaluation Questionnaire

A short evaluation questionnaire regarding ease of use and preference for each VAS method was given to each participant at the end of each measurement day. One participant failed to complete this questionnaire.

### Statistical Analysis

The statistical package used was SPSS version 16. Area Under the Curve data was initially analysed using ANCOVAs with age and gender as covariates in separate analyses. Neither age or gender were associated with VAS scores therefore were not controlled for in the final analyses. Repeated measures (3(method)*2(condition)*11(time)) ANOVA with Bonferroni corrected comparisons were conducted for ratings of Hunger, Fullness and Desire to Eat. Correlation coefficients were calculated for Area Under the Curve between P&P and EARS II. Bland & Altman tests [11] were used to assess the agreement between the different methods of conducting VAS.

## Results

### Method of VAS

**Figure 1:**
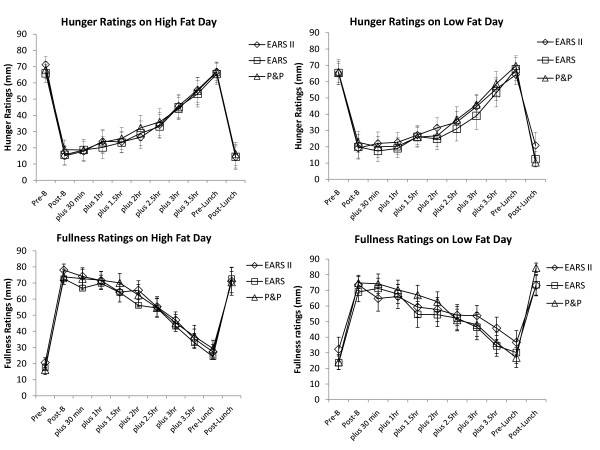
**Comparison of subjective hunger and fullness ratings using P&P, EARS and EARS II on the High Fat and Low Fat meal days**. Data shows mean (± SEM). Abbreviations on x-axis: Pre-B (Pre-Breakfast), Post-B (Post-Breakfast), plus 30 min (30 min after breakfast) continued until Pre-Lunch (4 hours post-breakfast) and Post-Lunch.

There was no significant difference between the three methods for Hunger, Fullness or Desire to Eat for either the high or low fat conditions (*p *= < 0.05) (See figure 1). There was a significant effect of time for hunger, fullness and desire to eat in all three methods (*p *= < 0.05) but no effect of high or low fat condition in any of these questions (p = 0.950, 0.997 and 0.729 respectively). Figure 1 shows the temporal profiles for hunger and fullness on both the high and low fat conditions. Profiles of desire to eat were similar to patterns observed for hunger.

### Direct Comparison - P&P and EARS II

**Figure 2:**
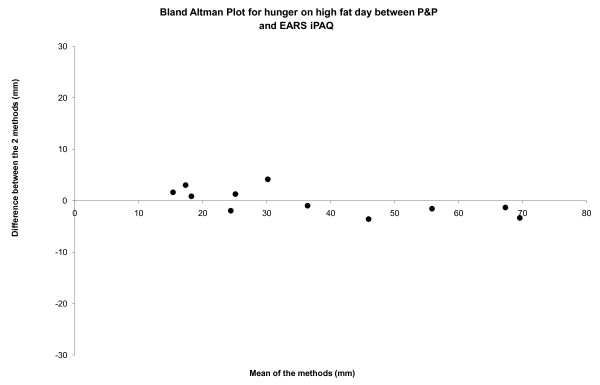
**Bland Altman plot showing agreement between traditional P&P and EARS II for hunger ratings on the HF meal day (95% CI = -1.10 to 3.14)**.

Correlation coefficients between P+P and EARS II for the high fat day, were performed on Area Under the Curve and found to be highly significant (table 1). Bland and Altman analyses revealed minimal levels of bias between P&P and EARS II with all points within 95% confidence interval (-1.10 to 3.14) (see figure 2).

**Table 1:** Correlation coefficients between P&P and EARS II for daily hunger, fullness and desire to eat on the high fat day

	R^2^	*P* value
**Hunger AUC**	0.89	<0.0001

**Fullness AUC**	0.96	<0.0001

**Desire to Eat AUC**	0.95	<0.0001

### Evaluation of method

An evaluation questionnaire was administered to assess ease of use and preference for a particular method. Out of 11 participants, 6 (55%) rated that P&P was the easiest to use, 5 (45%) chose EARS II, whereas none of the participants chose EARS as the easiest to use. In terms of preference, 3 (27%) preferred P&P and 8 (73%) preferred EARS II. Again, none of the participants chose EARS as the preferred method.

## Discussion

The present study was designed to assess the validity of a new technique for administering VAS electronically. There were no differences between the standard method - P&P - and either the previously validated EARS or the new method - EARS II. The methods did vary slightly, but there was no systematic pattern to the variation. That is, one method did not consistently produce higher or lower results. The sensitivity and oscillations in appetite sensations in response to the meals were similar between the three methods, even at the higher scores where differences are more likely to be seen [5]. However, it would not be advised to use these methods interchangeably since previous studies have found significant differences between P&P and EARS [8] that were not detected in this study. This may be due to the highly controlled nature of the present study exaggerating the similarities between the methods. On a practical note, participants involved in the study showed a preference for the EARS II. They also showed a dislike for the EARS because of the time delay in moving the cursor from the mid-point to either extreme of the line. Furthermore, since the vertical marker on the EARS is set to appear at 50 mm for each question, the participant could skip one or more questions without the researcher knowing if the data were valid. An added benefit of EARS II is that a vertical marker is only present once the participant responds to the question. In this method, skipped questions are coded to help the researcher identify missing data.

Limitations of using VAS to measure appetite have previously been identified. For example, the argument that hunger and other subjective measures of appetite are hypothetical constructs was identified many years ago, with the concern that people can perceive them in different ways [12]. It is also recognised that hunger is to an extent environmentally determined and seen as a product of time and context [1]. For example, when asking participants about hunger their first thought is often to recall when their last eating episode occurred and from that they may deduce whether they are hungry or not. However within subject designs as employed in the present study limit these extraneous effects and these issues should not vary between the VAS methods. A limitation of the present study is that we did not undertake a test-retest element to assess reliability and determine whether there were any differences between the new EARS II measures when taken on separate occasions. Additionally, the sample size used in the present study could be considered a limitation. Previous research has demonstrated that a sample size of N = 12 was sufficient to detect a within-subject difference >10 mm [13]. This criterion was deemed appropriate to detect systematic variation in ratings due to technical and physical differences between methods (e.g. screen size, response format, etc.). Due to the controlled, laboratory-based nature of this study, there were no missing data to consider. However, this will need to be addressed when validating the EARS II in free-living settings.

The present study evaluated the EARS II, which enables data capture in laboratory and free-living settings. This device allows the researcher to maintain an element of control over administration of measures. For example, through the use of reminder alarms and security- protected software that participants cannot breach. Since all data is date- and time-stamped, missing data can be easily identified and coded. The PC compatibility and versatility of EARS II mean the future uses of the system could include additional measures and continuous recording on one single PDA unit. In nutrition research there is a need for appetite to be accurately monitored under free-living conditions and for continuous monitoring in laboratory and free living settings over extended periods of time. With appropriate modifications there is the potential for single handheld units to measure self-reported food intake, physical activity records, food preference assessments and cognitive performance tests.

## Conclusions

The EARS II system was sensitive to the impact of a meal and recovery of appetite during the postprandial period. There was no difference between the P&P (traditional bench mark) method and either the EARS or EARS II systems. The EARS II is therefore a valid method for monitoring appetite sensations. In addition, participants expressed a preference for using EARS II. Since the EARS II offers increased versatility and the potential for future modification for use in clinical and free-living conditions, this study supports further validation of EARS II in larger, free-living studies.

## Competing interests

The authors declare that they have no competing interests.

## Authors' contributions

CG made contributions to study design, acquisition of data, analysis and interpretation of data and drafted the manuscript. PC aided study design, data collection and interpretation of data. GF conceived of the study, coordinated the analysis, helped in interpretation of data and drafted the manuscript. NK added to the interpretation and analysis of data and drafted the manuscript, in particular revising it critically for important intellectual content. JB helped conceive the study, interpreted the results and drafted the manuscript. All authors read and approved the final manuscript.
